# A new common mutation in the hemagglutinin of the 2009 (H1N1) influenza A virus

**DOI:** 10.1371/currents.RRN1162

**Published:** 2010-06-01

**Authors:** Sebastian Maurer-Stroh, Raphael Tze Chuen Lee, Frank Eisenhaber, Lin Cui, Shiau Pheng Phuah, Raymond TP Lin

**Affiliations:** ^*^Bioinformatics Institute (BII), A*STAR Singapore; ^†^Bioinformatics Institute (BII), A*STAR; ^‡^Bioinformatics Institute (BII) A*STAR Singapore and ^§^National Public Health Laboratory, Ministry of Health, Singapore

## Abstract

As the 2009 (H1N1) influenza A virus continues evolving, most mutations appear geographically and temporally confined. However, the latest surveillance data suggests emergence of a new prominent mutation, E391K, in the hemagglutinin (HA) that is globally on the rise. Interestingly, when modelled in the context of the available HA crystal structure, this mutation could alter salt bridge patterns and stability in a region of the HA oligomerization interface that is important for membrane fusion and also a known antigenic site. We discuss occurrence of HA-E391K in global surveillance data and associated clinical phenotypes from Singapore ranging from mostly mild to few severe symptoms, including sporadic vaccine failure. More clinical and experimental data are needed to determine if this mutation could alter the biology and fitness of the virus or if its increased occurrence is due to founder effects.

## Emergence of the HA-E391K mutation

The pandemic 2009 (H1N1) influenza A virus [1] [2] [3] continues to circulate worldwide, giving rise to concerns about new strains emerging which might cause further outbreaks. As a well-connected international hub, Singapore receives a broad sample of globally circulating influenza strains and 2009 (H1N1) remains the dominant influenza strain. The mutation HA-E391K was first identified in New York in July 2009 and appeared shortly after also in Singaporean samples but only grew rapidly in appearance recently (Figure 1). HA-E391K has been found in samples from 20 countries so far.

**Figure:**
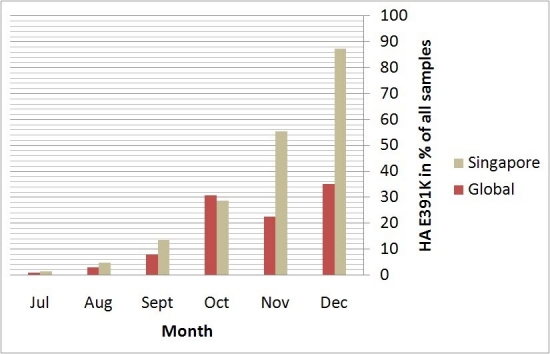
**Figure 1.** **Rise of the HA-E391K mutation globally and in Singapore.** **Data for global occurrence is based on sequences downloaded from the NCBI Influenza Virus Resource** [4]**** **for the respective time periods. All dates are based on collection date and as there is a natural delay between sample collection, sequencing and data deposition, the latest data with sufficient number of samples is from December 2009. Singapore samples and sequences are from National Public Health Laboratory, Ministry of Health Singapore.**

New substitutions that are globally found in more than 20% of the sequences with collection date December 2009 in the NCBI Influenza Virus Resource [4] include HA-D114N, HA-E391K, PA-V14I, PA-K716Q, PB1-K736G, PB1-R563K and PB2-K340N. Analysis of their co-occurrences identifies 9 clusters, 5 of which include HA-E391K (Table 1). All of these variants co-occur with the globally dominant strain (variant (ii) in Table 1) characterized by the 5 substitutions HA-S220T, NA-V106I, NA-N248D, NP-V100I and NS1-I123V. PB2-K340N is also of interest as it frequently co-occurred with the potentially virulent HA-D239G mutation [5] [6] but this may also be the reason for its increased occurrence due to a sampling bias towards sequencing more severe cases.

**Table:** 

**Variant**	**HA**	**NA**	**NP**	**NS1**	**PA**	**PB1**	**PB2**	**% circulating strains in Dec 2009**
i	Con.	V106I, N248D	V100I	Con.	Con.	Con.	Con.	0.8
ii	S220T	V106I, N248D	V100I	I123V	Con.	Con.	Con.	42.5
iii	S220T	V106I, N248D	V100I	I123V	Con.	Con.	K340N	15.0
iv	S220T	V106I, N248D	V100I	I123V	V14I	Con.	K340N	0.8
v	S220T, E391K	V106I, N248D	V100I	I123V	Con.	Con.	K340N	1.7
vi	D114N, S220T, E391K	V106I, N248D	V100I	I123V	V14I	R563K	Con.	0.8
vii	D114N, S220T, E391K	V106I, N248D	V100I	I123V	Con.	R563K	Con.	18.3
viii	S220T, E391K	V106I, N248D	V100I	I123V	V14I, K716Q	K736G	Con.	13.3
ix	S220T, E391K	V106I, N248D	V100I	I123V	Con.	Con.	Con.	6.7

**Table 1. Co-circulating variants based on frequent marker mutations in December 2009. Residue numberings and mutations are relative to reference strain A/Texas/04/2009(H1N1). Con. … conserved.**

 Looking at the temporal detail of the variants including HA-E391K, the percentage of co-occurrences with PA-V14I, PA-K716Q and PB1-K736G has changed from September 2009 to December 2009 (56.3%, 68.0%, 57.4% and 34.8% respectively). The HA-E391K co-occurrences with HA-D114N and PB1-R563K on the other hand increased from September 2009 to December 2009 (6.3%, 26.7%, 39.3% to 47.8% respectively). This co-occurrence can be found both in Europe and the USA. The co-occurrences of HA-E391K with PB2-K340N was found only recently in 2 viral strains in December 2009 (one in Poland and the other in Greece). The co-occurrence of HA-E391K with HA-D114N, PB1-R563K and PA-V14I was found in December 2009 in one strain from Spain. Variants with HA-E391K but without the 6 other substitutions were also found in Germany, Nicaragua, Taiwan and the USA.

**Figure:**
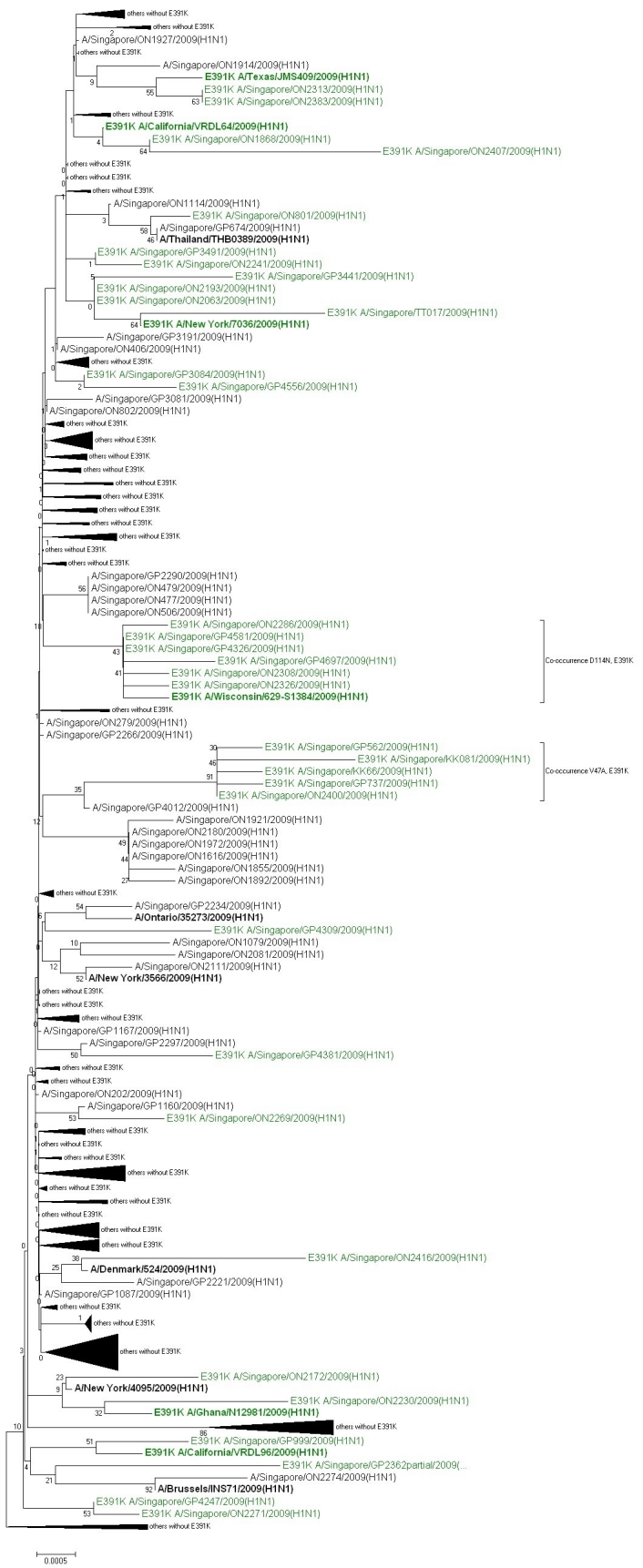
**Figure 2**. **Evolutionary relationships of 307 HA coding region nucleotide sequences from Singapore and their closest global matches as identified by BLAST [7]. Neighbor-Joining [8] consensus tree from 500 bootstrap replicates [9] using the Maximum Composite Likelihood method [10] to calculate evolutionary distances in units of the number of base substitutions per site. The rate variation among sites was modeled with a gamma distribution (shape parameter=2). All codon positions not containing gaps were included, leaving a total of 1691 positions in the final dataset. Phylogenetic analyses were conducted in MEGA4 [11]. Strains with HA-E391K are colored green, all non-Singaporean sequences are in bold letters. Subtrees are only shown in detail if they contain at least one HA-E391K sample. **

Phylogenetic analysis of the Singaporean sequences including their closest non-Singaporean international matches shows that sequences with HA-E391K have appeared multiple times in different contexts (Figure 2). While in some cases, the best match is another Singaporean sequence without HA-E391K, in others it is a non-Singaporean sequence already with HA-E391K which could indicate import from or exchange with other global outbreak clusters including HA-E391K. It should be noted, however, that detailed phylogenetic relations are difficult to conclude from this analysis due to the high sequence similarity among the strains. Other sequence clusters that include a second co-occurring mutation among all closely related strains in the respective subtree are found for HA-E391K with HA-D114N (see discussion of global appearance of these coupled mutations above) as well as HA-E391K with V47A which would be indicative of founder effects for these cases. The relative dominance of HA-E391K containing strains in Singapore compared to their average global occurrence in December 2009 (Figure 1) may have originated from founder effects.

## HA-E391K in the oligomer interface of the crystal structure

**(A)**

**Figure:**
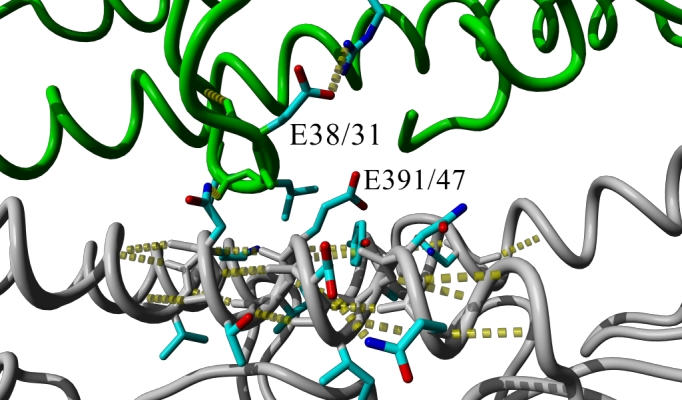

**(B)**

**Figure:**
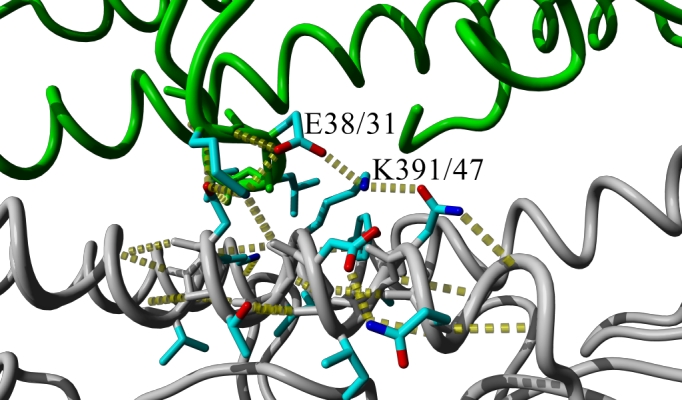
** Figure 3.  Hydrogen bond network (yellow dotted lines) at the oligomerization interface of two HA monomers (gray or green backbone, respectively) in the 2009 H1 crystal structure [12] with (A) wildtype E 391 (47 in HA2 in alternative numbering) or (B) mutant K 391 after simulated annealing energy minimization through short MD simulation using the AMBER force field as implemented in Yasara [13]. In the mutant, HA-E391K, the intra-molecular salt bridge of E38/31 to R336/321could be replaced by an inter-molecular salt bridge between E38/31 from one monomer to K391/47 in the other which weakens stability of the region within the monomer while it strengthens the interaction between the monomers.**

**Table:** 

****

The hemagglutinin position 391 corresponds to the HA2-subunit position 47 mentioned by Smeenk and Brown [14] [15]. In their studies with mouse-adapted H1N1 influenza virus variant A/FM/1/47, the W47G substitution causes a decrease in optimum pH of membrane fusion which led to an increase in necrosis of the bronchiolar epithelium, peribronchiolar lymphocytes, and airway obstruction. This effect can partially be explained mechanistically as the mutation drastically alters the oligomerization interface in a region that undergoes structural changes required for membrane fusion [16] [17]. As also identified by the authors of another HA crystal structure [18] published while this analysis was in preparation, the HA-E391K mutation alters this very same region. Additionally we find that the **intra**-molecular salt bridge of E38 to R336 could be broken to allow an **inter-**molecular salt bridge instead between E38 from one monomer to K391 in the other (Figure 3). This could weaken stability of the membrane fusion region within an individual monomer while it strengthens the interaction between the monomers. Furthermore, this region was recently identified as highly conserved epitope recognized by antibodies that neutralize the closely related 1918 H1N1 virus by blocking the structural changes associated with membrane fusion [19]. Ultimately, only experimental or detailed clinical data will be able to establish if HA-E391K has the potential to alter the biology of the virus-host interactions and antigenicity or if its increasing occurrence is due to founder effects.

## Clinical information for samples with E391K

The clinical phenotype for 35 Singaporean samples that had the HA-E391K mutation was available for analysis (Table 2). 

**Table:** 

**Identifier**	HA mutations* **^1^**	**Date received**	**Age**	**Vaccination Status**	**Symptoms**
ON801	N277D, E391K, V428I	7/1/2009		No	Mild
GP3084	E391K	8/18/2009	48	No	Mild
ON1868	E391K	8/22/2009	28	No	Mild
GP3441	I22V, S88P, E391K, I392S	8/31/2009	8	No	Mild
GP3491	E391K	9/1/2009	8	No	Mild
ON2018	E391K	9/4/2009	29	No	Mild
ON2063	E391K	9/10/2009	18	No	Mild
ON2083	V190I, E391K	9/13/2009		No	Mild
ON2090*^2^	E391K	9/14/2009		No	Mild
ON2172	V7I, N55D, E391K	10/1/2009		No	Mild
ON2193	E391K	10/8/2009		No	Mild
ON2230	N55D, E189K, E391K	10/19/2009	6	No	Mild
ON2241	E391K	10/21/2009	12	Unknown	Mild
ON2269	M274I, E391K	11/3/2009		Unknown	Mild
ON2271*^2^	E391K	11/3/2009	17	No	Mild
GP4247	E391K	11/6/2009	15	No	Mild
ON2286*^3^	D114N, E391K	11/10/2009	18	Unknown	Mild
GP4309	E391K	11/12/2009	5	No	Mild
GP4326	D114N, E391K	11/13/2009	30	Unknown	Mild
ON2308	D114N, E391K, K460E	11/16/2009	48	Unknown	Mild
ON2313	E391K	11/17/2009	26	Unknown	Mild
GP4381	K60E, S91R, E391K, R467K	11/18/2009	26	No	Mild
GP4556	E391K	12/1/2009	19	No	Mild
GP4581	D114N, E391K	12/2/2009	60	No	Mild
GP4697	D114N, F347L, E391K, R563K	12/10/2009	29	Unknown	Mild
ON2400*^3^	V47A, E391K	12/11/2009		Unknown	Mild
ON2407*^3^	I78V, E391K	12/13/2009	11	Unknown	Mild
ON2416	V36I, E391K	12/15/2009		Unknown	Mild
TT017	A302T, E391K, V544I	1/8/2010		Unknown	Mild
KK066	V47A, E391K	1/27/2010		Unknown	severe (ICU, death)
GP562	V47A, E391K	2/2/2010	19	No	Mild
KK081	V47A, A203V, E391K	2/12/2010		Unknown	severe (ICU, death)
GP737	V47A, K71N, E391K	2/12/2010	45	No	Mild
GP999	N142D, E391K	2/26/2010	12	Yes	Mild
GP2362 partial	N142D, E391K, E516K	5/18/2010	25	Yes	Mild

*^1^ relative to reference A/New York/20/2009(H1N1) which additionally differs from A/California/07/2009(H1N1) by the HA mutations P100S, S220T, I338V; *^2^ low reactor against A/California/07/2009(H1N1)-derived antisera; *^3^ normal reactor against A/California/07/2009(H1N1)-derived antisera.

**Table 2. Summary of HA mutations for patients with at least partial clinical info.**

 33 of the 35 cases had only mild symptoms, while 2 were severe (intensive care unit or death). Although this would result in 5.7% of cases with the mutation being severe, this number is likely to be heavily overestimated since all severe cases are systematically analyzed in Singapore while mild cases are mainly added through routine surveillance. The age distribution among patients with HA-E391K continues to follow the general pattern of 2009(H1N1) by strongly affecting the younger age groups. 

For the severe cases, no additional known mutation, such as HA-D239G (D222G or D225G in alternative numberings [5] [6]), was found that could directly explain severity. In both of the severe cases with HA-E391K, we also find a HA-V47A mutation which is structurally located in vicinity (13 Angstrom) of the E391K mutation (Figure 4) and, hence, could contribute to any effects in the region as discussed above. At the same time, there have been three other cases with both HA-E391K and HA-V47A but showing only mild symptoms. As  4 of the 5 cases with HA-E391K and HA-V47A were closely temporally related (within ~2 weeks), the co-occurrence could derive from the same transmission chain/cluster as also indicated by their grouping in the phylogenetic analysis.

**Figure:**
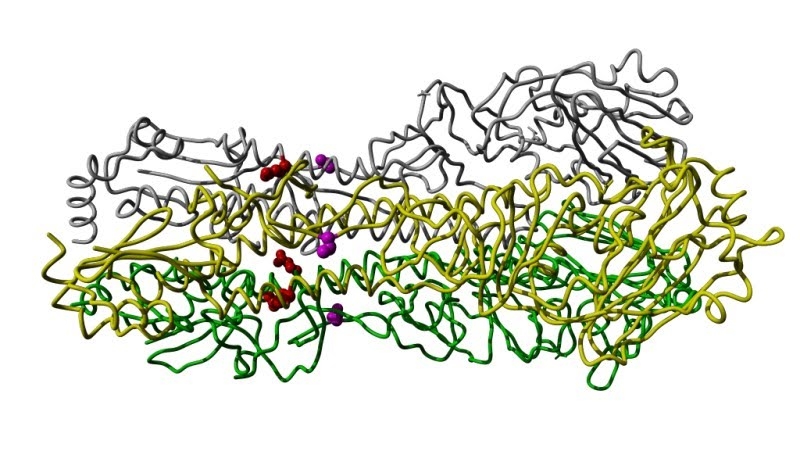
**Figure 4. Crystal structure** [12] **of the HA trimer (monomers in gray, yellow or green backbone). The residues involving mutations E391K (position 47 in HA2 in alternative numbering) and V47A (position 40 in HA1 in alternative numbering) are shown as balls and colored red or magenta, respectively.**

Initially, the HA-E391K mutation came to our attention as it occurred in a special case where a patient had been vaccinated but still contracted 2009(H1N1) as evidenced by sequencing. Besides HA-E391K, the sample additionally had the mutation HA-N142D which was previously recognized as antibody escape mutant in the context of H5-type viruses (N142D corresponds to N129D in H3 numbering in Table 2 of reference [20]). Interestingly, a recent second vaccine failure case showed the same co-occurrence of  HA-E391K and HA-N142D. Hence, it is not clear if the vaccine failure in these cases was due to HA-E391K, HA-N142D or patient-specific factors. 

For 5 samples of 2009(H1N1) with HA-E391K, reaction towards the vaccine based on strain A/California/07/2009(H1N1) was tested through hemagglutination inhibition assays by the regional WHO collaborating centre in Melbourne. Interestingly, 2 of the 5 samples were reported as low reactor. Although the currently dominant strain is more similar to the early genomic variant A/New York/20/2009(H1N1) than to the common vaccine strain A/California/07/2009(H1N1), these two strains have been previously found to be antigenically closely related by Garten et al. [1]. Consequently, if the common variations between dominant and vaccine strain do not alter antigenicity, the only additional mutation shared among the low reacting samples was HA-E391K. However, it should be noted that 3 of the 5 samples with HA-E391K (one with both HA-E391K and HA-V47A) still showed normal A/California/07/2009(H1N1)-like antigenicity.

Overall, these examples are interesting but not sufficient to significantly link the HA-E391K mutation (with or without HA-V47A) to increased severity or lower vaccine efficacy as this may depend on many other patient-specific factors and the fact that these cases had the mutation could readily be explained by the fact that strains with HA-E391K are currently the most common flavor among all locally available samples.

## Conclusion

HA-E391K is a globally fast growing mutation in 2009 (H1N1) samples and could alter the salt bridge pattern and stability in a region of the HA oligomerization interface that is important for membrane fusion and also a known antigenic site. More clinical and experimental data are needed to determine if this mutation could alter the biology and fitness of the virus or if its increased occurrence is due to founder effects.

## Acknowledgments

The authors wish to thank Michael Levitt for constructive discussions regarding the salt bridges involving HA-E391K and the regional WHO collaborating centre in Melbourne for HI testing. Some of the early Singaporean samples downloaded from Genbank were sequenced by the Genome Institute Singapore (GIS) for which we would like to acknowledge Christopher Wong and Martin Hibberd.

## Funding Sources

NPHL is funded by the Ministry of Health, Singapore and BII by the Agency for Science Technology and Research (A*STAR), Singapore.

## Competing Interests

The authors declare that no competing interests exist. 
